# Changing Tactics? Optimizing ECT in difficult-to-treat depression (ChaT): study protocol

**DOI:** 10.1192/j.eurpsy.2024.291

**Published:** 2024-08-27

**Authors:** L. Van den Eynde, K. Vansteelandt, K. Hebbrecht, J. Obbels, S. Verspecht, L. Gistelinck, P.-J. Geerts, D. Schrijvers, P. Sienaert

**Affiliations:** ^1^Academic Center for ECT and Neuromodulation (AcCENT), UPC KU Leuven, Kortenberg; ^2^Psychiatry, AZ Sint Jan Bruges, Bruges; ^3^Psychiatry, AZ Groeninge, Kortrijk; ^4^Collaborative Antwerp Psychiatric Research Institute (CAPRI), UAntwerpen, Antwerp, Belgium

## Abstract

**Introduction:**

Electroconvulsive therapy (ECT) is an evidence-based treatment for difficult-to-treat depression, in which an electrical stimulus is applied via right unilateral (RUL) (Fig 1) or bitemporal (BT) electrodes (Fig 2). Current guidelines recommend to start ECT with RUL placement, except for cases where rapid response is needed. BT ECT has the reputation of exerting a stronger and faster antidepressive effect, but is associated with more pronounced cognitive side effects, as compared to RUL ECT. Recent studies, however, suggest comparable outcomes. In patients responding to ECT, most of the improvement in depressive symptom severity is witnessed early in the treatment course. In case of non-response, it is common practice to switch from RUL to BT electrode placement, although scientific evidence is lacking. As an answer to this research gap, the ChaT-trial was designed: a randomized controlled trial (RCT) to address which treatment strategy (either continue RUL ECT or switch to BT ECT) speeds up recovery with the least impact on cognitive function, in case of early non-response after 4 ECT sessions.

**Objectives:**

1. To compare the antidepressant efficacy and cognitive effects of continuing RUL ECT vs switching to BT ECT.
2. To assess group and subject-specific trajectories of depressive symptom severity and neurocognitive performance during the acute ECT course and up to 3 months post-treatment.

**Methods:**

This multi-center double-blind RCT includes adult patients with a uni- or bipolar depression. In case of non-response (<50% decrease of IDS-CR score (Inventory of Depressive Symptomatology-Clinician Rated)) after 4 sessions of brief-pulse high-dose RUL ECT, patients are randomized to either continue RUL ECT, or switch to brief-pulse moderate dose BT ECT until remission. Depressive symptoms are assessed by IDS-CR, Psychotic Depression Assessment Scale (PDAS) and CORE assessment of psychomotor change. An extensive neuropsychological test battery is used to assess different domains of cognitive functioning, e.g., autobiographical memory using the Colombia University- Autobiographical Memory Interview Short- Form (CU-AMI-SF)(Fig 3).

**Results:**

Our hypotheses are: (1) continuing RUL ECT is non-inferior to switching to BT ECT in terms of depressive symptom severity, and (2) continuing RUL ECT is superior to switching to BT ECT in terms of cognitive side effects.

**Image:**

**Figure:**
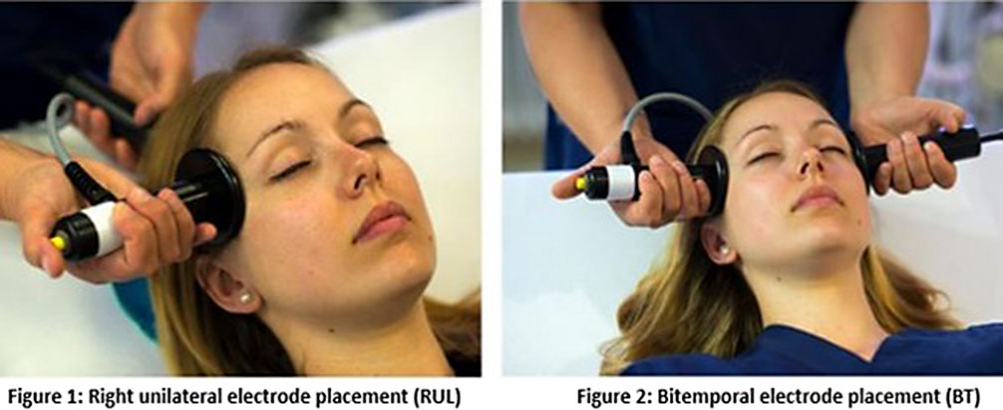

**Image 2:**

**Figure:**
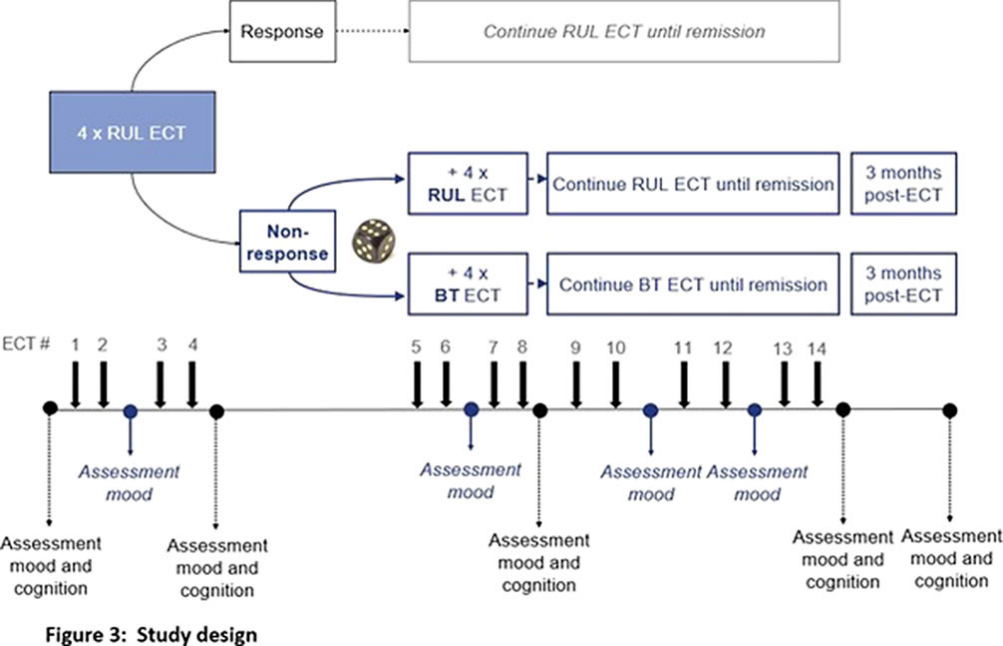

**Conclusions:**

The ChaT-trial is the first RCT comparing antidepressant efficacy and cognitive effects of continuing RUL ECT with switching to BT ECT in case of early non-response during an acute ECT-course. The results may optimize clinical decision making, speeding up recovery, while minimizing cognitive side effects.

**Disclosure of Interest:**

None Declared

